# Effects of percussive therapy dosages on recovery from acute exercise-induced muscle damage: a systematic review and meta-analysis

**DOI:** 10.1186/s12998-026-00657-9

**Published:** 2026-06-12

**Authors:** Yang Zhu, Lele Yang, Tao Liu, Fuya Yao, Qilong Wang, Zheng Yi

**Affiliations:** Institute of Physical Education and Training, Capital University of Physical Education and Sports, Beijing, China

**Keywords:** Percussive therapy, Muscle strength, Post-exercise recovery, Explosive performance, Meta-analysis, Delayed onset muscle soreness, Physical training

## Abstract

**Objective:**

Percussive therapy (PT) is increasingly used for post-exercise recovery, but its effects and dose-related responses after exercise-induced muscle damage remain uncertain. This review evaluated the effects of PT on neuromuscular performance, muscle soreness, and biochemical markers after acute exercise.

**Methods:**

The study adhered to PRISMA guidelines. A comprehensive search was conducted across PubMed, Embase, Web of Science, the Cochrane Library, and CNKI for Randomized Controlled Trials (RCTs) published through November 26, 2025. Risk of bias, methodological quality, and certainty of evidence were assessed using RoB 2, PEDro, and GRADE. Data analysis was performed using Stata-MP 18.0 software.

**Results:**

Twelve RCTs were included. PT improved countermovement jump recovery compared with control (k = 13, g = 0.78, 95% CI 0.26 to 1.29, *p* < 0.01; low certainty) and reduced creatine kinase levels (k = 7, g = − 0.87, 95% CI − 1.57 to − 0.17, *p* = 0.02; very low certainty). PT did not clearly improve maximum voluntary contraction recovery (k = 8, g = 0.12, 95% CI − 0.10 to 0.34, *p* = 0.28; moderate certainty) or delayed onset muscle soreness (k = 17, g = 0.14, 95% CI − 0.19 to 0.48, *p* = 0.40; very low certainty). Exploratory analyses suggested that longer treatment (> 5 min per muscle group) was associated with larger countermovement jump effects, whereas 2.5–5 min protocols at higher frequency (≥ 50 Hz) were associated with creatine kinase reduction. Longer treatment was also associated with higher delayed onset muscle soreness scores.

**Conclusion:**

PT may improve explosive performance recovery and reduce early creatine kinase levels, but current evidence does not support clear benefits for maximum strength recovery or soreness relief. Dose-related findings are preliminary and should not be interpreted as prescriptive thresholds.

**Supplementary Information:**

The online version contains supplementary material available at 10.1186/s12998-026-00657-9.

## Introduction

Skeletal muscle is fundamental to human movement and is highly susceptible to mechanical stress. Strenuous eccentric exercise or unaccustomed high-intensity exercise often causes exercise-induced muscle damage (EIMD) [1]. This damage begins with mechanical disruption of the muscle fiber ultrastructure and may subsequently trigger excitation–contraction coupling impairment, calcium imbalance, and inflammatory responses [2]. Clinical manifestations include leakage of muscle enzymes, such as creatine kinase (CK), a common indirect marker of post-EIMD sarcolemmal disruption, and delayed onset muscle soreness (DOMS) [3, 4]. More importantly, neuromuscular performance suffers acute impairment. This appears as a sustained decline in Maximum Voluntary Contraction (MVC) and Countermovement Jump (CMJ) performance [5]. Failure to recover from this functional decline significantly affects training schedules and exercise experience. It also increases the risk of injury [6]. Functional limitations caused by EIMD hinder continuous high-intensity activity. This applies to both elite athletes and the general active population [7]. Traditional recovery strategies (such as cold therapy and static stretching) often require specific equipment or professional assistance [8]. Handheld Percussive Therapy (PT) emerged to overcome these limitations. PT delivers rapid, continuous mechanical pulses to deep muscle tissue. It has become a highly popular method for acute recovery [9].

PT is a widely used recovery modality that combines localized vibration and repetitive mechanical stimulation applied to soft tissue [10–12]. Although several physiological mechanisms have been proposed, including changes in local blood flow, pain modulation, tissue stiffness, and tissue fluid exchange, direct evidence supporting these mechanisms remains limited [13, 14]. Therefore, the current literature is more informative regarding whether PT produces measurable recovery effects than regarding the exact mechanisms by which these effects occur. Original trials have more consistently shown short-term improvements in ROM, whereas effects on strength and post-exercise recovery remain less consistent. For example, 5-min PT protocols have been reported to increase ankle or posterior-shoulder ROM without clear strength enhancement [15, 16]. Regarding post-exercise recovery, Alves et al. [17] reported reduced DOMS, fatigue, and perceived recovery after long-distance running but no improvement in vertical jump performance, whereas Roberts et al. [18] observed only transient pain relief without sustained improvement during the 24–72 h recovery period. Similarly, Leabeater et al. [19] found limited effects of a 5-min massage-gun treatment on physical and perceptual recovery after strenuous calf exercise. In contrast, [20, 21] reported favorable effects on strength-related recovery, with moderate-to-large effect estimates in some outcomes. Previous PT-specific systematic reviews have examined massage-gun/PT effects on performance, recovery, flexibility, and pain, but these reviews were broad syntheses rather than dose-focused quantitative reviews of acute EIMD recovery [13, 14]. Taken together, the current evidence suggests a possible divergence between subjective symptom relief and objective functional recovery.

This substantial heterogeneity may be partly explained by unstandardized intervention dosages (e.g., treatment duration, frequency, amplitude, and pressure). PT outcomes are likely influenced by dose-related parameters; however, current protocols remain heterogeneous and evidence-based guidance is still limited [12, 22]. Previous PT-specific systematic reviews have summarized the literature, but they were largely qualitative and also noted important methodological and reporting limitations in the available studies [13, 14].

Therefore, this systematic review and meta-analysis aimed to quantitatively evaluate the effects of PT on MVC, CMJ, CK, and DOMS after acute exercise. Particular emphasis was placed on whether dosage parameters, including treatment duration, frequency, and amplitude when available, were associated with differences in recovery outcomes. We hypothesized that PT would show outcome-specific effects after acute exercise and that variation in dosage parameters might partly explain heterogeneity across studies. This review may provide a more evidence-based and cautious basis for interpreting PT as a post-exercise recovery strategy.

## Materials and methods

### Registration

This systematic review and meta-analysis was conducted in accordance with PRISMA 2020 and the methodological recommendations of the Cochrane Handbook for Systematic Reviews of Interventions [23]. Before the formal literature search, study selection, and data extraction, the protocol was prospectively registered in PROSPERO (CRD420251240832).

### Search strategy

**Fig. 1:**
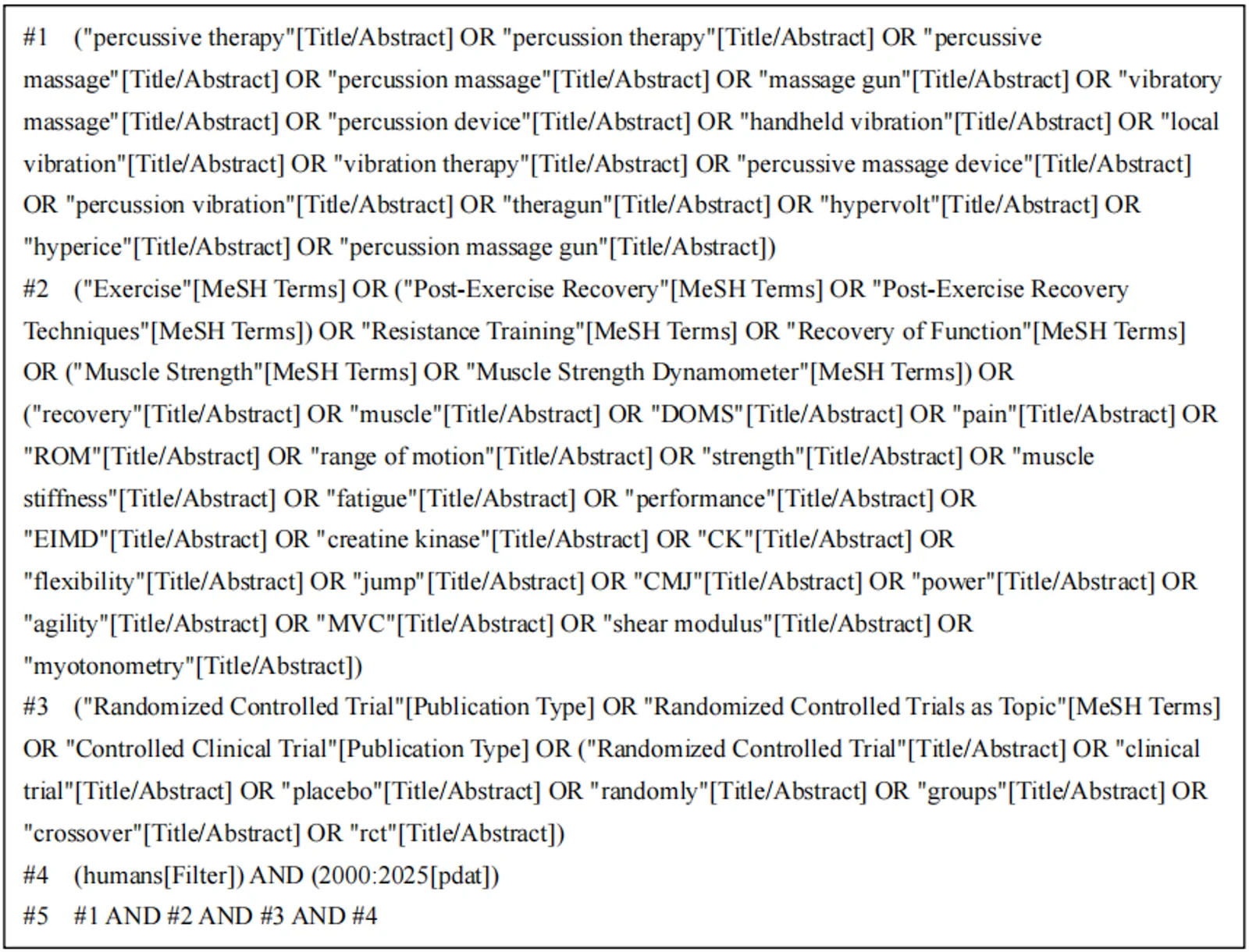
PubMed search formula

Electronic databases, including PubMed, Web of Science, Cochrane Library, Embase, and CNKI, were searched for relevant studies published between 2000 and November 26, 2025. The year 2000 was selected as the start date because this review focused on modern handheld percussive devices/massage guns rather than occupational vibration exposure, whole-body vibration, or non-handheld vibration therapy. Additional studies were identified by manually screening the reference lists of eligible articles and relevant reviews. The search strategy combined controlled vocabulary terms (e.g., MeSH terms) with free-text keywords. The PubMed search strategy is shown in Fig. 1, and detailed search strings for the other databases are provided in Supplementary Table 1.

### Inclusion and exclusion criteria

Inclusion and exclusion criteria for the literature were established based on the PICOS (Participants, Interventions, Comparisons, Outcomes, and Study Design) principles [24].

#### Inclusion criteria

1. Participants: Healthy adolescents or adults, including physically active individuals, recreational athletes, or athletes, who were free from recent illness or musculoskeletal injury.
2. Intervention: Local mechanical stimulation restricted to massage guns, percussive massage, or high-amplitude local vibration. The intervention was required to be administered as a single session within 1-h post-exercise.
3. Comparison: The control group received passive recovery (rest) or a placebo intervention.
4. Outcomes: Studies had to report baseline values and at least one follow-up assessment within 72 h after the intervention for DOMS, CK, MVC, or CMJ. This window was chosen because DOMS and functional impairments after EIMD typically emerge and peak within 24–72 h, representing the acute recovery phase [4].
5. Study Design: Randomized controlled trials (RCTs) or randomized crossover trials with an adequate washout period.

#### Exclusion criteria

1. Studies involving clinical populations, current musculoskeletal injury, or medication/supplement use likely to influence muscle recovery.
2. Studies combining PT with another active recovery modality (e.g., PT + massage vs massage) were excluded because such comparisons estimate the add-on effect of PT under a co-intervention rather than the stand-alone effect of PT, reducing the interpretability of PT-specific dosage effects [25].
3. Studies using non-handheld vibration, whole-body vibration, occupational vibration exposure, manual massage alone, foam rolling alone, or other interventions not meeting the operational definition of PT.
4. Studies with insufficient data for extraction or where clarification from the corresponding authors was unobtainable.
5. Duplicate publications or overlapping datasets.

### Literature screening

Search results were managed using EndNote X9 software, with duplicates removed by an independent reviewer (YZ). Subsequently, two reviewers (LL, TL) independently screened titles and abstracts against the inclusion criteria. Studies failing to meet eligibility standards were excluded, and full-text articles were retrieved for detailed evaluation. Any discrepancies were resolved through discussion to reach consensus, with a third reviewer (ZY) adjudicating if necessary. Two reviewers independently extracted and cross-checked the data; inconsistencies were verified by a third reviewer. In instances of missing or incomplete data, corresponding authors were contacted to ensure data integrity. Inter-reviewer agreement at the full-text stage was quantified using Cohen’s κ [26].

### Data extraction

Data extraction was performed independently by two researchers utilizing a standardized Microsoft Excel template. The following parameters were extracted: (1) Basic characteristics: First author, publication date, study type, and participant details (sample size, gender, age, BMI). (2) Study design: Exercise type and protocol, PT protocol, outcome measures, and monitoring time points. For studies presenting results exclusively in graphical format, data were extracted using WebPlotDigitizer-4.8[27].

### Quality assessment

Methodological quality and risk of bias were assessed separately by two independent reviewers (LL and TL), with disagreements resolved by discussion or adjudication by a third reviewer (ZY).

#### Methodological quality

Methodological quality was summarized using the Physiotherapy Evidence Database (PEDro) scale [28]. Items 2–11 contributed to the total score, with scores ≥ 6, 4–5, and ≤ 3 interpreted as high, moderate, and low quality, respectively. PEDro was used as a descriptive summary of trial methodological quality in physiotherapy research and did not replace the domain-based RoB 2 assessment.

#### Risk of bias within studies

Risk of bias was assessed using the revised Cochrane risk-of-bias tool for randomized trials (RoB 2) [29]. RoB 2 was used as the primary domain-based assessment of internal validity. The five domains were the randomization process, deviations from intended interventions, missing outcome data, measurement of the outcome, and selection of the reported result. Overall judgments were classified as low risk, some concerns, or high risk. Risk-of-bias plots were generated using the robvis Shiny web app [30].

### Statistical analysis

All statistical analyses were performed using Stata-MP 18.0 software. A quantitative synthesis (meta-analysis) was performed only when a minimum of two included studies reported data for the same continuous outcome measure. To minimize bias arising from baseline differences, “change scores” (mean and standard deviation) were prioritized as input data for effect sizes. Given that outcomes were continuous variables measured using diverse tools across studies, Hedges’ g (corrected Standardized Mean Difference, SMD [31]) and 95% Confidence Intervals (95% CI) were selected as the summary statistics. Descriptive statistics for continuous variables were presented as mean ± standard deviation (mean ± SD). Effect sizes were interpreted according to Cohen's guidelines [32]: < 0.2 indicating trivial, 0.2–0.5 small, 0.5–0.8 moderate, and > 0.8 large effects. Heterogeneity was assessed using Cochran’s Q test and the I2 statistic. Significant heterogeneity was defined as *p* < 0.10 or I2 > 50%, in which case a random-effects model was employed; otherwise, a fixed-effect model was applied. Subgroup analyses and meta-regression were conducted to explore sources of heterogeneity and potential dose–response relationships. The following covariates were analyzed: (1) Duration per muscle group (≤ 2.5 min, 2.5–5 min, > 5 min); (2) PT frequency (< 50 Hz, ≥ 50 Hz); (3) Monitoring time points (0 h, 24 h, 48 h, 72 h post-intervention); and (4) Exercise type (concentric, eccentric, aerobic). Sensitivity analysis was performed using the “leave-one-out” method [33], which involved removing one study at a time and recalculating the pooled effect size to verify result stability. Publication bias was assessed visually using funnel plots, followed by Egger’s regression test for quantitative assessment [34]. Where potential bias was identified, Duval and Tweedie’s “Trim and Fill” method was applied for correction [35]. The significance level was set at *p* < 0.05 for all tests, with the exception of heterogeneity tests (*p* < 0.10). All statistical tests were two-sided.

### GRADE evidence assessment

The certainty of evidence for the primary outcomes was assessed independently by two reviewers (TL and LL) using the GRADE framework [36].Because all included studies were randomized controlled trials, the initial certainty was rated as high and then downgraded when appropriate for risk of bias, inconsistency, indirectness, imprecision, or publication bias. Disagreements were resolved by discussion or adjudication by a third reviewer (ZY). The final certainty was classified as high, moderate, low, or very low.

## Results

### Search results

**Fig. 2:**
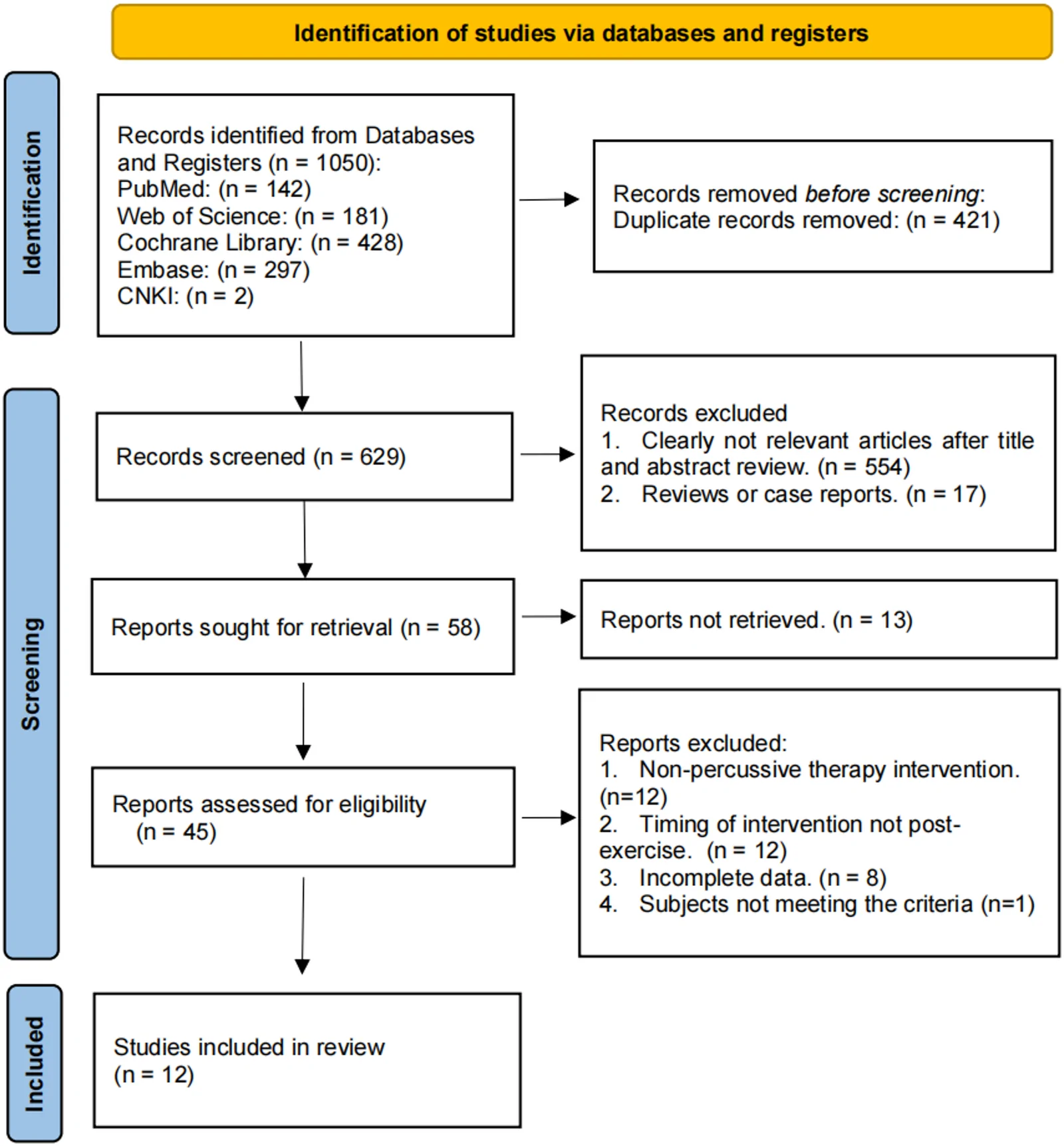
Flow diagram of the selection process

The initial database search yielded a total of 1,050 records (PubMed, n = 142; Web of Science, n = 181; Cochrane Library, n = 428; Embase, n = 297; CNKI, n = 2). Following removal of 421 duplicates using EndNote X9, 629 records were screened by title and abstract (Cohen’s κ = 0.908), and 571 records were excluded. Fifty-eight reports were sought for retrieval; 13 reports could not be retrieved because full text was unavailable. The remaining 45 full-text reports were assessed for eligibility (Cohen’s κ = 0.880; see Supplementary Material S2), of which 33 were excluded for failing to meet the inclusion criteria. Ultimately, 12 studies met the eligibility criteria and were included in the quantitative synthesis (Fig. 2).

### Characteristics of included studies

The study included 12 trials published between 2023 and 2025. The total sample size was 393 participants (286 males and 107 females). The largest sample size was 84 [17], and the smallest was 10 [8]. Ages ranged from 17.6 to 37 years. All included studies were Randomized Controlled Trials (RCTs). Eleven were parallel-group RCTs, and one was a randomized crossover trial [19]. The crossover study used a split-body design. It used the contralateral limb as the control. Although there was no washout period, the anatomical separation distinguished the intervention effects. This design effectively controlled for individual differences. Therefore, it met our criteria for local muscle recovery, and we included it. Only one study involved athletes [37]. The rest involved healthy participants. Regarding the PT method, 11 studies used a “massage gun”. Only Wei et al. [8] named the intervention "vibration therapy." However, they used a Deep Muscle Stimulator (DMS). This device uses a piston-based mechanism and a frequency of 60 Hz. This fits the definition of PT, so the study was included. All studies reported treatment duration and frequency. However, data on amplitude was incomplete, so we did not analyze it. We categorized exercise types into concentric (5 studies [19, 21, 38, 39]), eccentric (6 studies [8, 18, 37, 40–42]), and aerobic exercise (1 study [17]). No adverse events were reported in the included studies (Tables 1, 2).

**Table 1:** Basic Information of the included studies

Included studies	Country of origin	Research Type	Research participant	Age (years)	Sample size		BMI (kg/m^2^)
					PT	CON	
Szajkowski [38]	Poland	PS	healthy adults	33.8 ± 3.2	11M/9F	11M/9F	24.37 ± 2.94
Alves [17]	Brazil	PS	Recreational runners	34.4 ± 1.5	24M/15F	32M/13F	25.47 ± 4.41
Li [39]	China	PS	healthy adults	21.5 ± 2.0	10M/10M	10M	21.70 ± 2.40
Wei [8]	China	PS	healthy adults	21.4 ± 1.6	5M	5M	22.64 ± 2.18
Ye [40]	American	PS	healthy adults	23.5 ± 3.8	7M/3F	6M/4F	25.89 ± 4.50
Chen [21]	China	PS	healthy adults	NR	20M	20M	NR
Heinke [41]	German	PS	healthy adults	23.8 ± 3.9	8M/3F	9M/3F	24.95 ± 5.53
Sarac [37]	Turkey	PS	athletes	20.3 ± 1.7	12M	12M	23.7 ± 1.6
Leabeater [19]	Australia	CS	healthy adults	21.3 ± 1.4	31M/34F	31M/34F	24.5 ± 2.6
Roberts [18]	American	PS	healthy adults	23.9 ± 2.7	2M/7F	1M/7F	25.9 ± 4.9
Dai [20]	China	PS	healthy participants	18.75 ± 1.05	10M	10M	20.80 ± 2.20
Kong [42]	China	PS	healthy adults	20.92 ± 0.80	10M	10M	22.80 ± 2.55

PT: Percussive Therapy intervention group; CON: Control group; PS: Randomized parallel-group trial; CS: Randomized crossover trial; M: Male; F: Female; NR: Not reported

**Table 2:** Interventional strategies and outcome indicators of the included studies

Included studies	Exercise plan	Intervention program		Outcome indicator	Monitoring time point
		PT	CON		
Szajkowski [38]	Single-leg calf raises to failure (4 sets, 1 min rest)	Massage gun (Hydragun); 53 Hz (3200 rpm); Soft attachment; 5 min total duration	Passive rest	DOMS	24h
Alves [17]	6.5–7.0 km road race (habitual running)	Massage gun (Avanutri GUN01); 55 Hz; 12 mm amplitude; Ball attachment; 10 min (Quadriceps)	Placebo	DOMS; CMJ	0h;24h;48h;72h
Li [39]	Weighted squats (60% 1RM, 10 sets × 7 reps, 2 min rest)	Massage gun (OUTSO 06); 53 Hz; 6 mm amplitude; Soft attachment.S-PMT: 25 min total (2.5 min/site); L-PMT: 40 min total (4 min/site)	Passive rest	DOMS; CMJ	0h;24h
Wei [8]	Weighted (10 kg) squat jumps and half-squat jumps (450 total reps, 10 sets, 2 min rest)	Local vibration (Deep Muscle Stimulator, DMS); 60 Hz; 10 min (3 min/site)	No intervention	CK; CMJ	0h;24h;48h;72h
Ye [40]	Eccentric elbow flexion (80% 1RM, 6 sets × 10 reps, 2 min rest)	Percussion massage gun (Therapist Select Prime, HoMedics); 47 Hz; 12 mm amplitude; 5 min total	No intervention	DOMS; MVC	0h;24h
Chen [21]	Knee extension (60% 1RM, 10 sets × 15 reps to failure, 2 min rest)	Massage gun; 40 Hz; Large ball attachment; 6 min total (2 min each for Vastus Lateralis, Rectus Femoris, and Vastus Medialis)	No intervention	MVC	0h;24h;48h
Heinke [41]	15-min downhill running followed by drop jumps to failure	Handheld percussive massage (Hypervolt 2); 40 Hz; Hard ball attachment; 8 min total	Supine rest	DOMS; CMJ	0h;24h;48h;72h
Sarac [37]	Combination of step-ups, drop jumps (50 cm), squats, and vertical jumps (100 continuous reps to induce DOMS)	Massage gun (TheraGun G2PRO); 40 Hz; Bilateral Quadriceps (5 min/side, 10 min total)	Supine rest	DOMS	24h;48h
Leabeater [19]	Bilateral heel raises on a 30 cm step (3 sets × 20 reps, 1 min rest)	Massage gun (Hydragun); 53 Hz; 5 min total (Medial Gastrocnemius 2.5 min + Lateral Gastrocnemius 2.5 min)	Passive rest	DOMS; CMJ	24h;48h
Roberts [18]	Unilateral eccentric dumbbell curls (85% 1RM, 6 sets × 10 reps, 2 min rest)	Massage gun (Hypervolt 2); 40 Hz; 12 mm amplitude; 1 min total	Passive rest	DOMS	24h
Dai [20]	Seated leg extension (60% 1RM, 10 sets × 15 reps, 2 min rest)	Massage gun (Hypervolt); Ball attachment; 40 Hz; Quadriceps (Rectus Femoris/Medialis/Lateralis); 5 min/leg (10 min total)	Supine rest	MVC; CMJ	0h;24h;48h
Kong [42]	Forward frog jumps and stationary squat jumps (10 sets × [20 + 20] reps, 2 min rest)	Massage gun (PHOENIX); 40 Hz; Vastus Medialis/Rectus Femoris/Vastus Lateralis; 5 min/muscle (15 min total)	No intervention	DOMS; MVC	24h

PT: Percussive Therapy intervention group; CON: Control group

### Quality assessment results

#### Risk of bias results

**Fig. 3:**
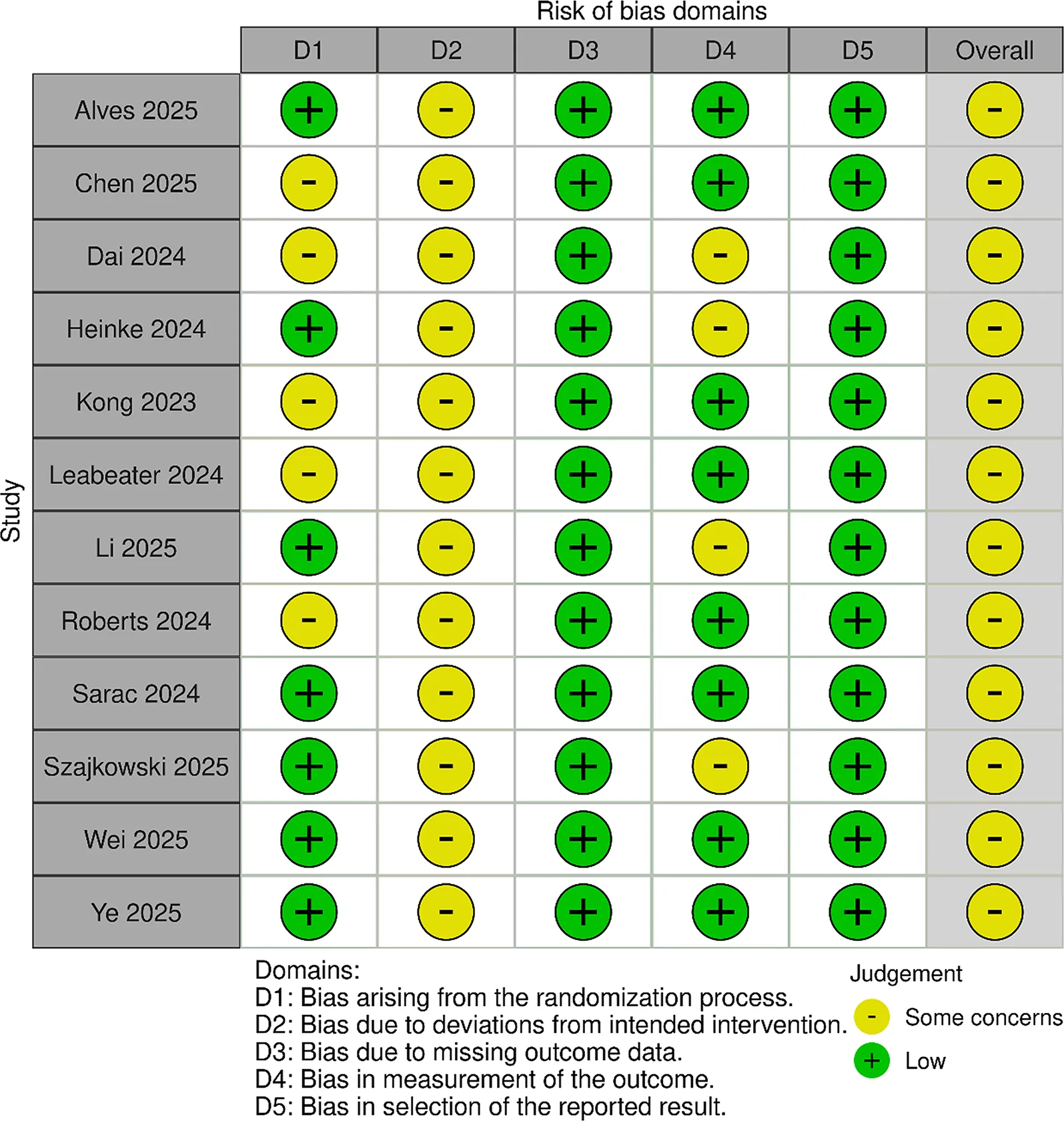
Risk of bias graph for included studies

**Fig. 4:**
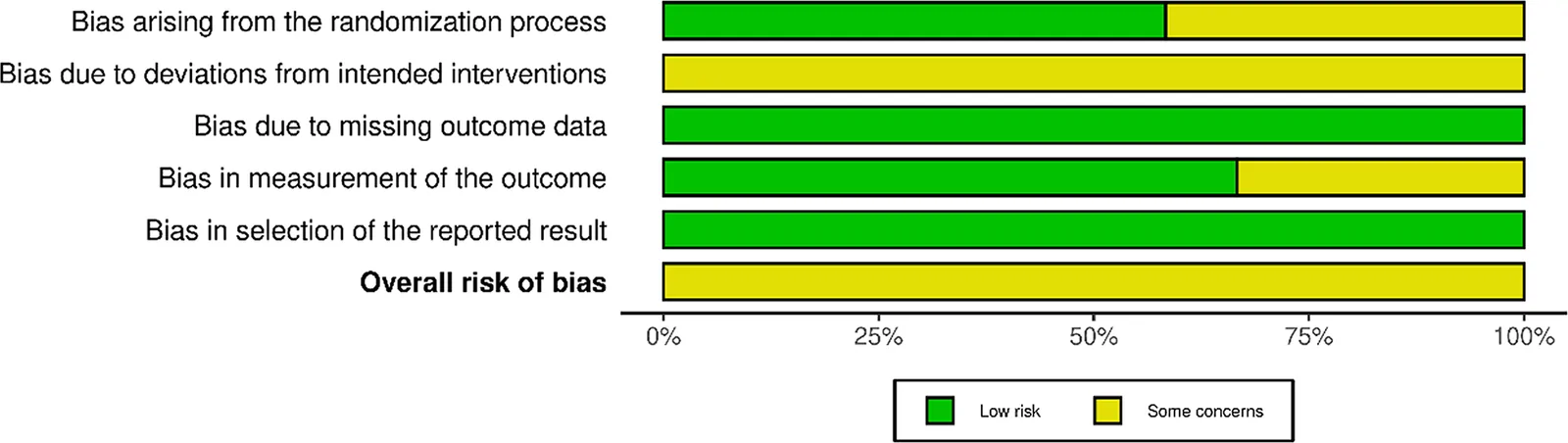
Summary of bias risk in included studies

Initial inter-rater agreement across the five RoB 2 domains ranged from substantial to almost perfect (Cohen’s κ = 0.65–1.00; see Supplementary Material). After consensus, all included studies were judged as having “some concerns,” and no study was rated as “high risk” (Figs. 3 and 4). Concerns were mainly related to deviations from intended interventions because participant and therapist blinding is difficult in PT trials, with additional concerns in randomization or outcome measurement in some trials. Intention-to-treat reporting was considered within the relevant RoB 2 domains but did not override domain-level concerns.

#### Methodological quality results

PEDro scores ranged from 6 to 8 (Table 3), with almost perfect inter-rater agreement (Cohen’s κ = 0.930). According to the prespecified thresholds, the included studies showed generally high methodological quality. Score reductions mainly reflected the difficulty of blinding participants and therapists in sensory physical interventions. PEDro scores were used descriptively and did not replace the domain-based RoB 2 judgments. Therefore, the included trials had acceptable methodological quality overall, but the findings should still be interpreted cautiously because blinding-related and domain-specific bias concerns remained.

**Table 3:** Quality assessment of included literature

Included studies	1	2	3	4	5	6	7	8	9	10	11	Total score
Szajkowski [38]	1	1	0	1	0	0	0	1	1	1	1	6
Alves [17]	1	1	1	0	0	0	1	1	1	1	1	7
Li [39]	1	1	1	1	0	0	0	1	1	1	1	7
Wei [8]	1	1	1	1	0	0	1	1	1	1	1	8
Ye [40]	1	1	1	1	0	0	1	1	1	1	1	8
Chen [21]	1	1	0	1	0	0	0	1	1	1	1	6
Heinke [41]	1	1	0	1	0	0	0	1	1	1	1	6
Sarac [37]	1	1	1	1	0	0	1	1	1	1	1	8
Leabeater [19]	1	1	0	1	0	0	0	1	1	1	1	6
Roberts [18]	1	1	0	1	0	0	0	1	1	1	1	6
Dai [20]	1	1	0	1	0	0	0	1	1	1	1	6
Kong [42]	1	1	0	1	0	0	0	1	1	1	1	6

1 denotes subject eligibility; 2 denotes random allocation; 3 denotes allocation concealment; 4 denotes baseline similarity;5 denotes subject blinding; 6 denotes clinician blinding; 7 denotes assessor blinding; 8 denotes dropout rate < 15%; 9 denotes intention-to-treat analysis; 10 denotes between-group statistical analysis; 11 denotes point measurements and difference values

### Meta-analysis results

#### MVC

**Fig. 5:**
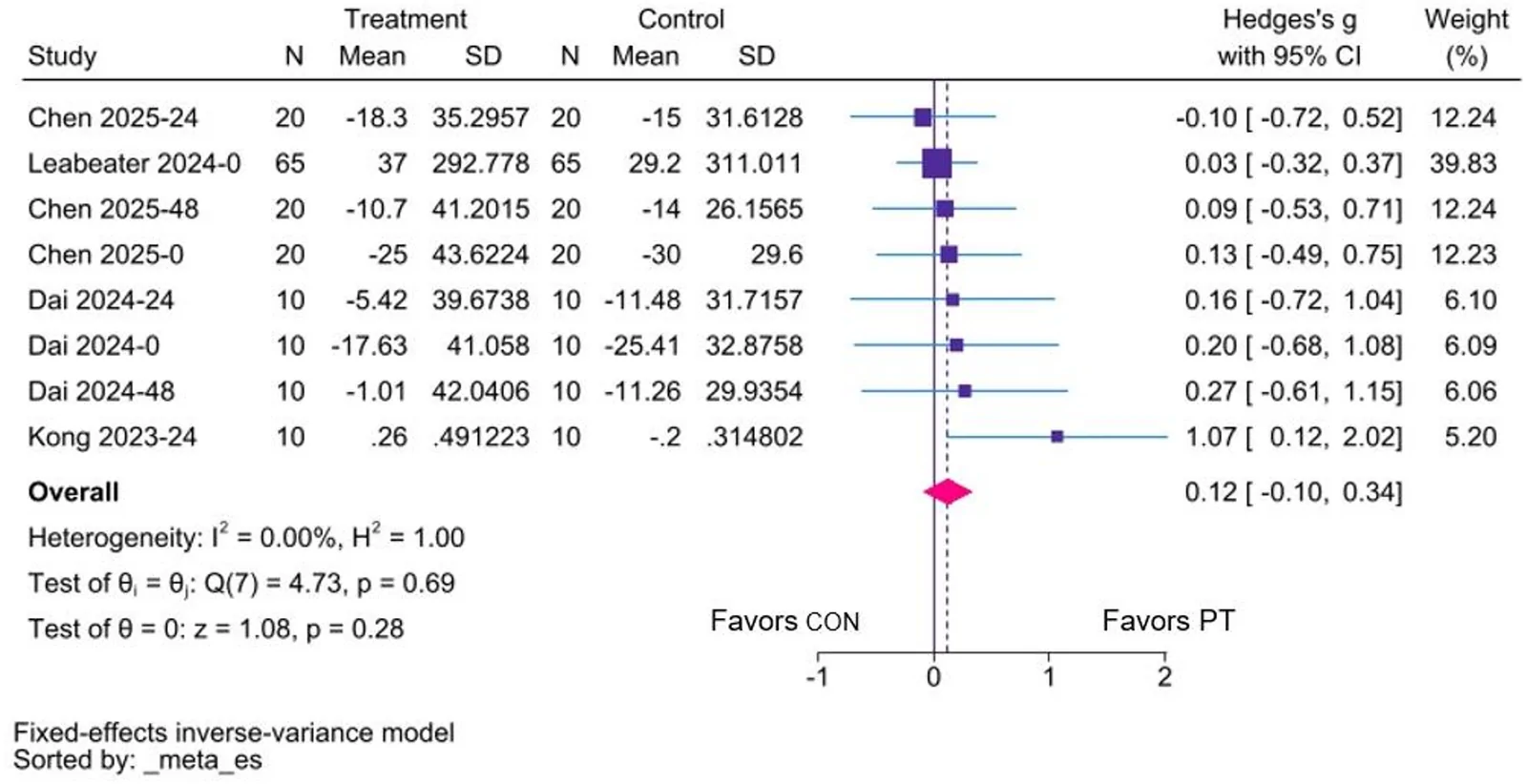
Meta-analysis forest plot of percussive therapy's effect on post-exercise MVC

A total of eight effect sizes were synthesized to evaluate the impact of PT on the recovery of muscle strength (MVC) following acute exercise. No statistical heterogeneity was observed among the studies (I2 = 0.00%, *P* = 0.69). The fixed-effect model showed that PT did not significantly improve MVC recovery compared to the control group (Hedges' g = 0.12, 95% CI − 0.10 to 0.34, *p* = 0.28) (Fig. 5).

Subgroup analysis indicated that exercise mode was a potential moderator (P_m_ = 0.04). However, only one study used eccentric exercise (k = 1). This difference was likely a "statistical artifact" caused by small sample bias [43]. Therefore, current evidence is insufficient to confirm this moderating effect. Duration per muscle group did not show a significant difference between groups (P_m_ = 0.16). However, the 2.5–5 min subgroup showed a trend toward improvement (g = 0.40, *p* = 0.08). This was better than the <2.5 min subgroup (g = 0.03). Furthermore, PT frequency (P_m_ = 0.49) and monitoring time points (Pm = 0.83) did not show significant moderating effects (Table 4).

**Table 4:** Subgroup Analysis of Percussive Therapy's Effect on Post-Exercise MVC

Subgroup	K(N)	Hedges'g	95%CI	P_d_	Q	I^2^(%)	P_m_
Duration of treatment							0.16
≤ 2.5min	4(85)	0.03	− 0.21 ~ 0.28	0.79	0.96	0.00	
2.5-5min	4(40)	0.40	− 0.05 ~ 0.85	0.08	0.48	0.00	
> 5min	NR						
Frequency							0.49
< 50Hz	7(80)	0.18	− 0.10 ~ 0.46	0.20	0.64	0.00	
≥ 50Hz	1(149)	0.03	− 0.32 ~ 0.37	0.88	N/A	N/A	
Monitoring time							0.83
0h	3(125)	0.07	− 0.22 ~ 0.35	0.65	0.91	0.00	
24h	3(80)	0.23	− 0.22 ~ 0.67	0.32	0.13	50.84	
48h	2(60)	0.15	− 0.36 ~ 0.66	0.56	0.15	0.00	
72h	NR						
Exercise Modality							0.04
Concentric	7(125)	0.07	− 0.16 ~ 0.29	0.55	0.99	0.00	
Eccentric	1(20)	1.07	0.12 ~ 2.02	0.03	N/A	N/A	
Aerobic	NR						

K(N): number of included effect sizes (total number of participants); P_d_: *p*-value of the pooled effect size for the subgroup; Q and I^2^(%): statistics for heterogeneity; P_m_: *p*-value for the test of subgroup differences. N/A: stands for not applicable. NR: No relevant information available

#### CMJ

**Fig. 6:**
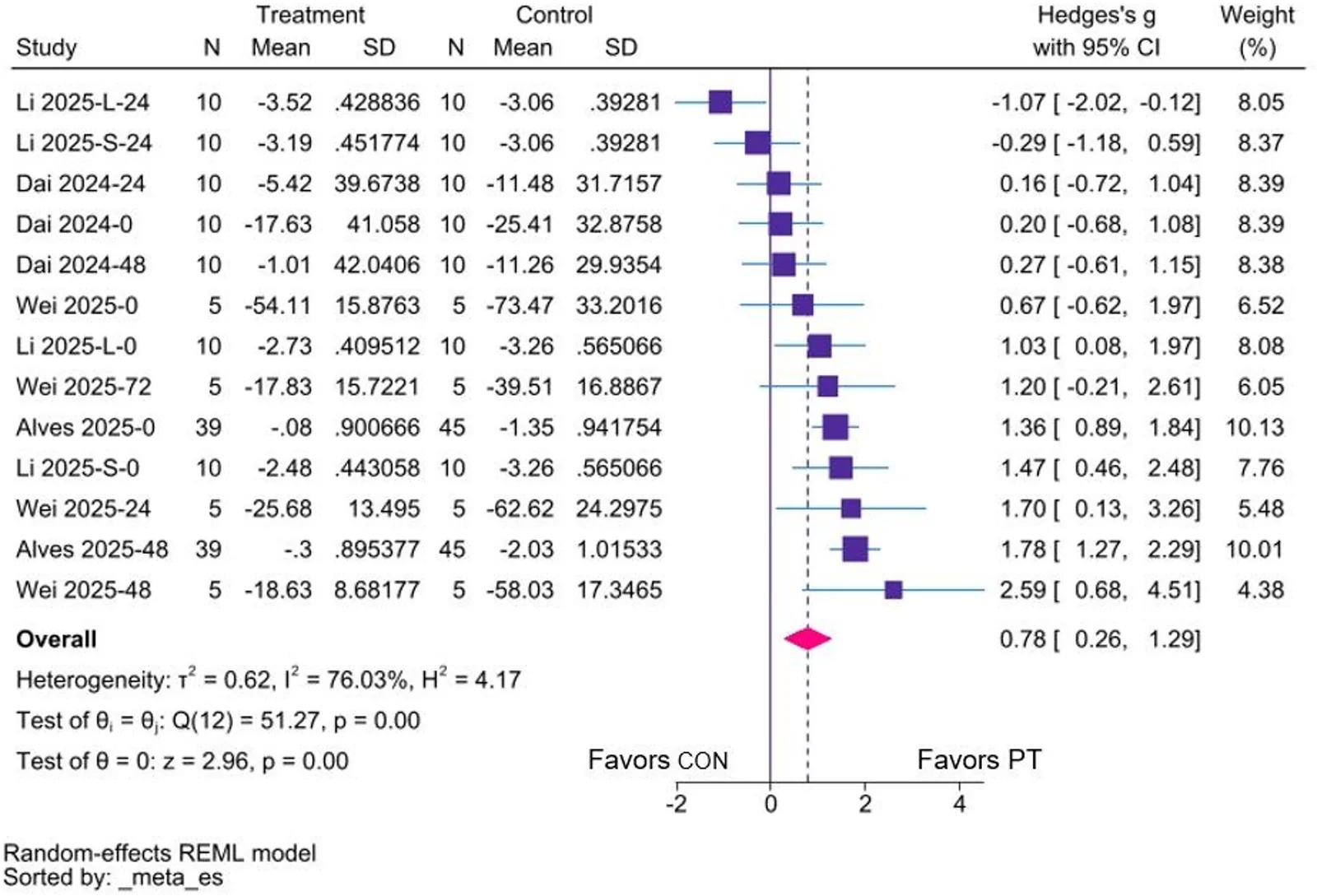
Meta-analysis forest plot of percussive therapy's effect on post-exercise CMJ

A total of 13 effect sizes were synthesized to evaluate the efficacy of PT on the recovery of explosive power (CMJ) following acute exercise. Significant heterogeneity was observed among the studies (I2 = 76.03%, *P*<0.01). The random-effects model showed that PT significantly promoted CMJ recovery compared to the control group. It showed a moderate effect size (Hedges' g = 0.78, 95% CI 0.26 to 1.29, *p* < 0.01) (Fig. 6).

Subgroup analysis and meta-regression showed that duration per muscle group and exercise mode were the main sources of heterogeneity. Regarding duration per muscle group, the difference between groups was significant (P_m_ = 0.02). Interventions > 5 min were the most effective (g = 1.56). They were significantly better than the ≤ 2.5 min and 2.5–5 min subgroups (both g = 0.57). However, meta-regression for continuous variables found no significant linear correlation between duration and effect size (*p* = 0.182). This suggests a "threshold effect." The effect increases significantly after a certain duration (> 5 min) rather than growing linearly. Regarding exercise mode, the difference between groups was significant (P_m_ < 0.01). Subgroup analysis showed that recovery after aerobic (g = 1.56) and eccentric exercise (g = 1.35) was significantly better than after concentric exercise (g = 0.24). Meta-regression confirmed this. Exercise mode was a significant predictor of effect size (β = 0.71, *p* = 0.002). Furthermore, PT frequency (subgroup P_m_ = 0.07, regression *p* = 0.200) and monitoring time points (subgroup P_m_ = 0.24, regression *p* = 0.657) did not show significant moderating effects (Table 5, Fig. 10).

**Table 5:** Subgroup Analysis of Percussive Therapy's Effect on Post-Exercise CMJ

Subgroup	K(N)	Hedges'g	95%CI	P_d_	Q	I^2^(%)	P_m_
Duration of treatment							0.02
≤ 2.5min	2(20)	0.57	− 1.16 ~ 2.30	0.52	0.01	84.91	
2.5-5min	9(50)	0.57	− 0.04 ~ 1.17	0.07	0.01	62.51	
> 5min	2(84)	1.56	1.15 ~ 1.97	0.00	0.24	27.61	
Frequency							0.07
< 50Hz	3(20)	0.21	− 0.30 ~ 0.72	0.42	0.99	0.00	
≥ 50Hz	10(134)	0.98	0.33 ~ 1.62	0.00	0.00	79.78	
Monitoring time							0.24
0h	5(134)	1.02	0.53 ~ 1.50	0.00	0.19	38.05	
24h	4(50)	− 0.00	− 0.99 ~ 0.98	0.99	0.02	73.12	
48h	3(114)	1.41	0.15 ~ 2.66	0.03	0.01	80.58	
72h	1(10)	1.20	− 0.21 ~ 2.61	N/A	N/A	N/A	
Exercise Modality							0.00
Concentric	7(40)	0.24	− 0.35 ~ 0.83	0.43	0.01	65.96	
Eccentric	4(10)	1.35	0.60 ~ 2.10	0.00	0.41	0.00	
Aerobic	2(84)	1.56	1.15 ~ 1.97	0.00	0.24	27.61	

K(N): number of included effect sizes (total number of participants); P_d_: *p*-value of the pooled effect size for the subgroup; Q and I^2^(%): statistics for heterogeneity; P_m_: *p*-value for the test of subgroup differences. N/A: stands for not applicable

#### CK

**Fig. 7:**
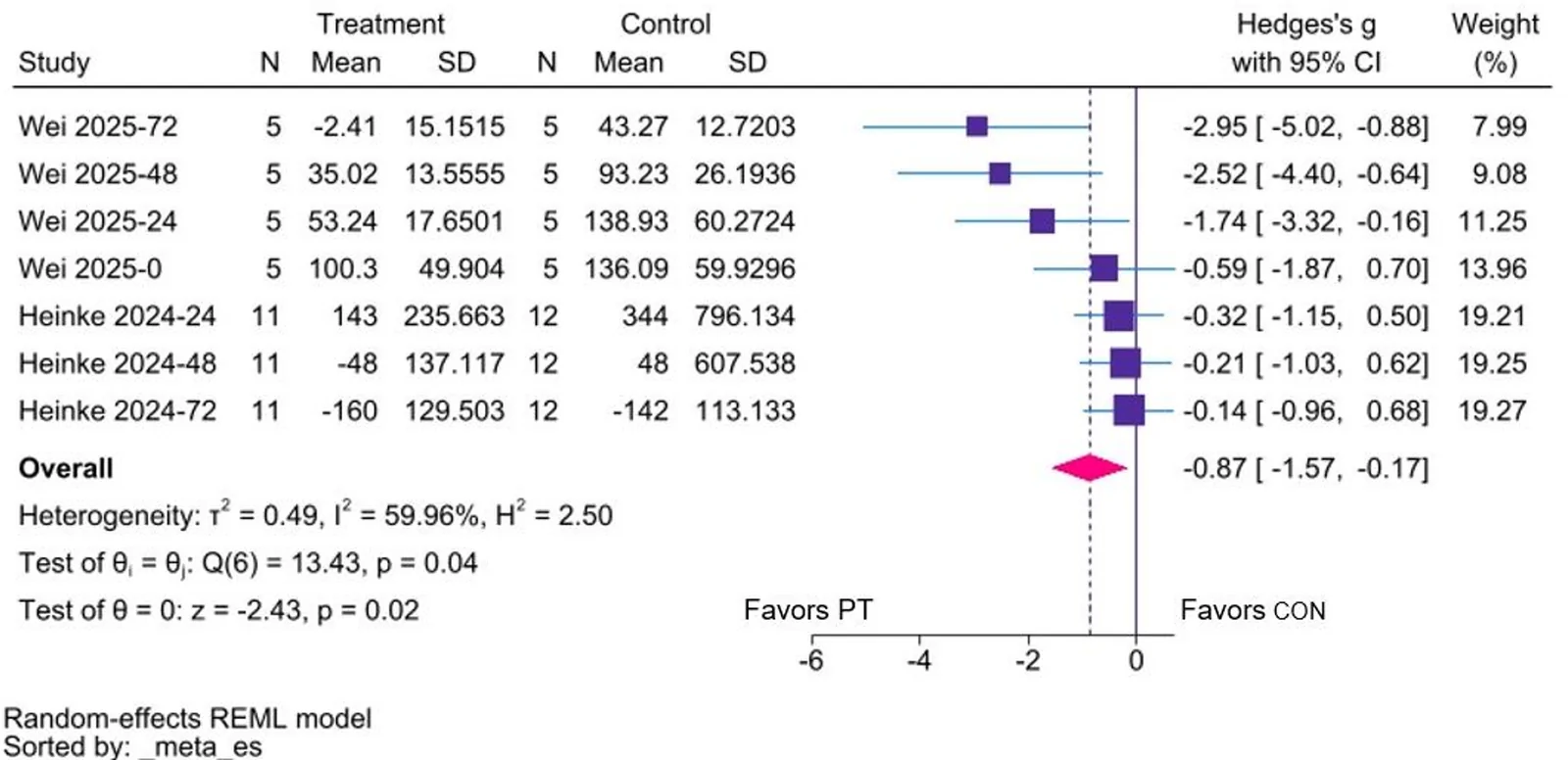
Meta-analysis forest plot of percussive therapy's effect on post-exercise CK

A total of seven effect sizes were synthesized to evaluate the impact of PT on CK levels following acute exercise. Moderate heterogeneity was observed among the studies (I2 = 59.96%, *P* = 0.04). The random-effects model showed that PT significantly reduced CK levels after acute exercise compared to the control group. It showed a large effect size (Hedges' g = − 0.87, 95% CI − 1.57 to − 0.17, *p* = 0.02) (Fig. 7).

Subgroup analysis indicated that duration per muscle group and PT frequency were the main sources of heterogeneity. Regarding duration per muscle group, the difference between groups was significant (P_m_ < 0.01). The 2.5–5 min intervention produced a significant reduction in CK (g = − 1.61, *p* < 0.01). However, interventions > 5 min did not show statistical significance (g = − 0.22, *p* = 0.36). PT frequency also showed significant differences between groups (P_m_ < 0.01). High-frequency PT (≥ 50 Hz) showed a significant effect (g = − 1.61, *p* < 0.01). Low-frequency PT (< 50 Hz) showed no significant effect. Monitoring time points did not show a significant moderating effect (P_m_ = 1.00). There were no statistical differences between effect sizes at different time points (0h, 24 h, 48 h, 72 h). Due to the limited number of included studies, we did not perform subgroup analysis for exercise mode (Table 6).

**Table 6:** Subgroup Analysis of Percussive Therapy's Effect on Post-Exercise CK

Subgroup	K(N)	Hedges'g	95%CI	P_d_	Q	I^2^(%)	P_m_
Duration of treatment							0.00
≤ 2.5min	NR						
2.5-5min	4(10)	-1.61	-2.42 ~ -0.80	0.00	0.17	39.68	
> 5min	3(23)	-0.22	-0.70 ~ 0.25	0.36	0.95	0.00	
Frequency							0.00
< 50Hz	3(23)	-0.22	-0.70 ~ 0.25	0.36	0.95	0.00	
≥ 50Hz	4(10)	-1.61	-2.42 ~ -0.80	0.00	0.17	39.68	
Monitoring time							1.00
0h	1(10)	-0.59	-1.87 ~ 0.70	0.37	N/A	N/A	
24h	2(33)	-0.63	-1.36 ~ 0.10	0.09	0.12	58.87	
48h	2(33)	-0.57	-1.33 ~ 0.18	0.13	0.03	79.47	
72h	2(33)	-0.52	-1.28 ~ 0.24	0.18	0.01	83.58	
Exercise modality							N/A
Concentric	NR						
Eccentric	7(33)	-0.58	-0.99 ~ -0.17	0.01	0.04	55.32	
Aerobic	NR						

K(N): number of included effect sizes (total number of participants); P_d_: p-value of the pooled effect size for the subgroup; Q and I^2^(%): statistics for heterogeneity; P_m_: p-value for the test of subgroup differences. N/A: stands for not applicable.NR: No relevant information available

#### DOMS

**Fig. 8:**
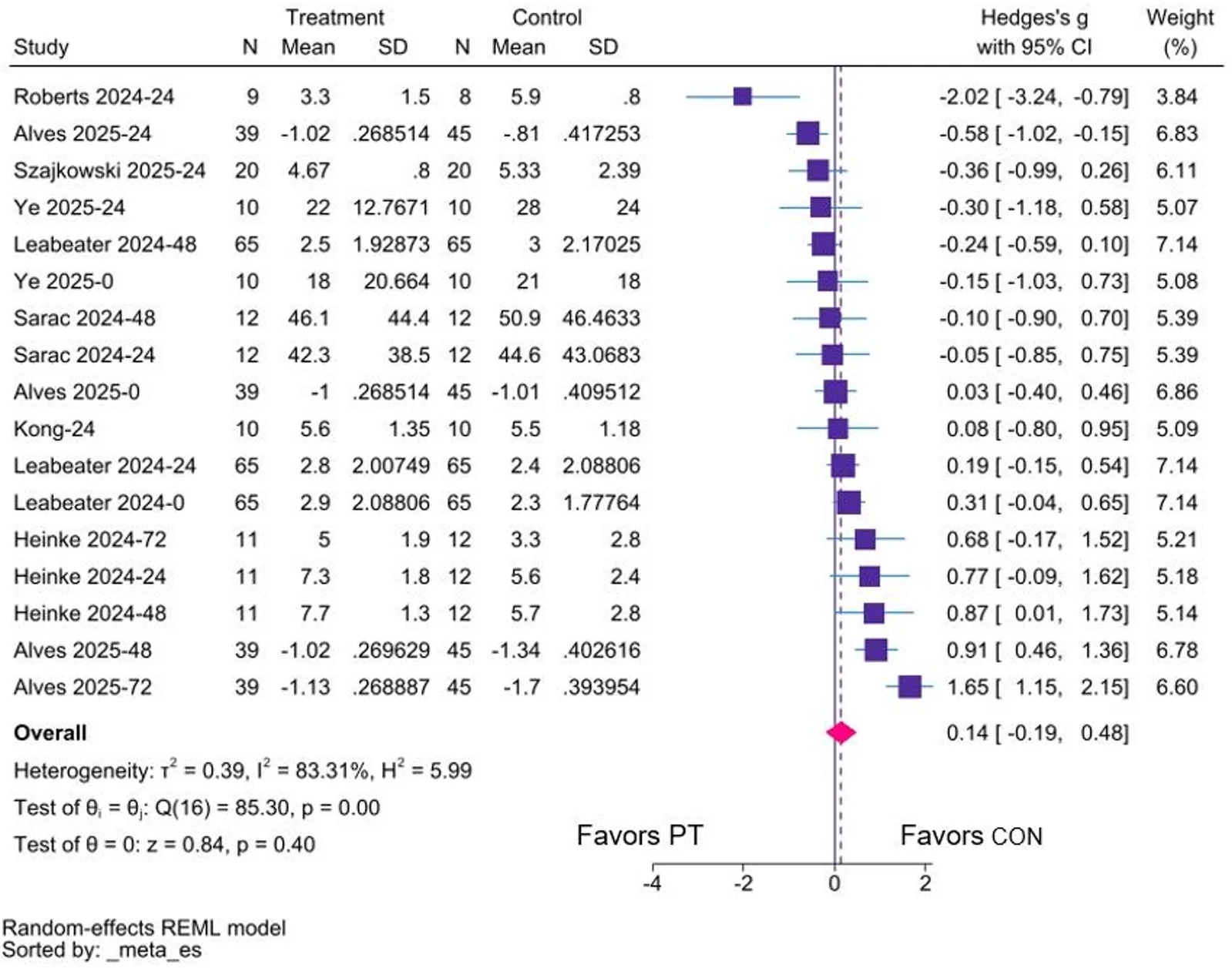
Meta-analysis forest plot of percussive therapy's effect on post-exercise DOMS

A total of 17 effect sizes were synthesized to evaluate the impact of PT on DOMS following acute exercise. High heterogeneity was observed among the studies (I2 = 83.31%, *P* < 0.01). The random-effects model showed that PT did not significantly reduce post-exercise DOMS compared to the control group (Hedges' g = 0.14, 95% CI − 0.19 to 0.48, *P* = 0.40) (Fig. 8).

**Fig. 9:**
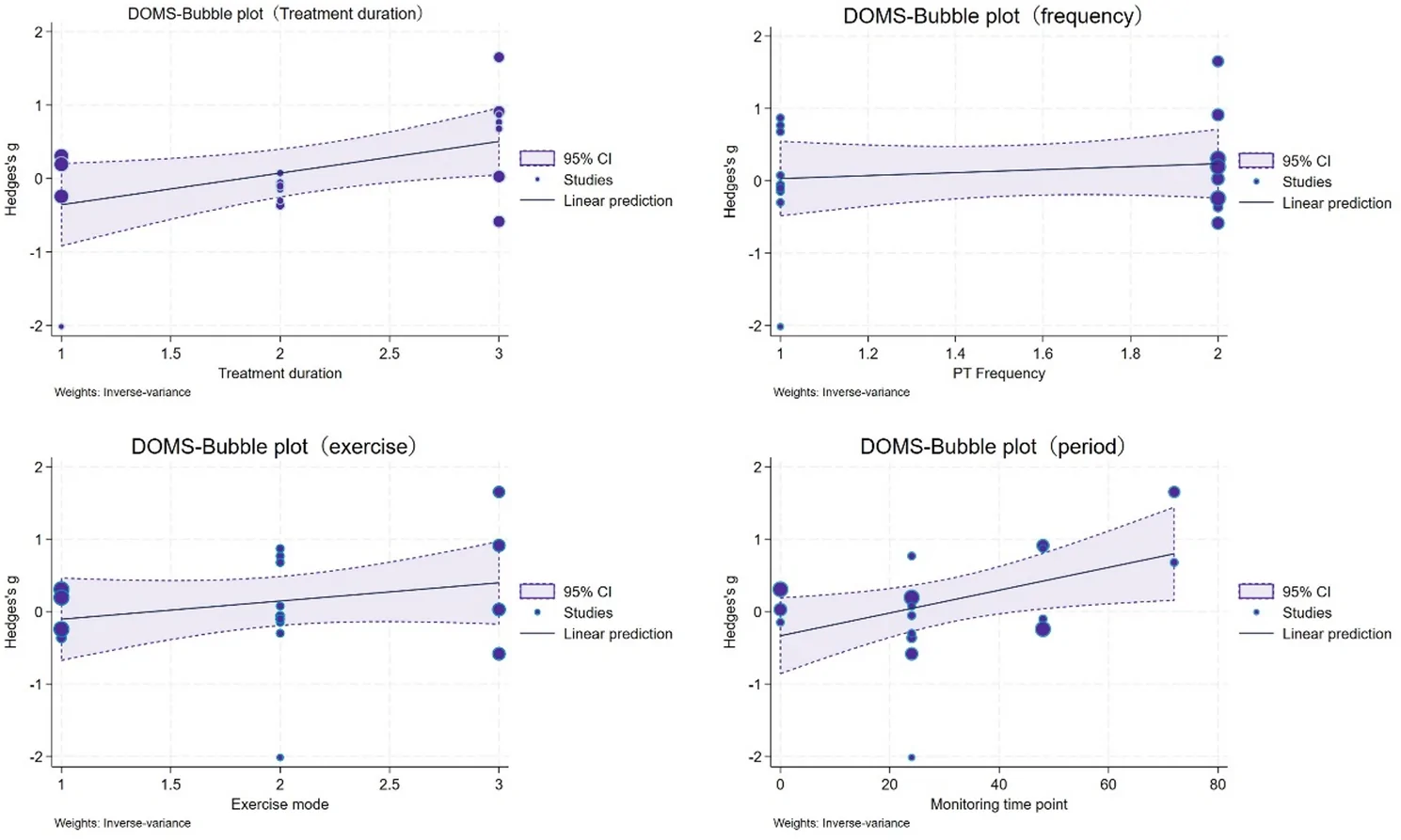
Meta-regression analysis of key variables affecting DOMS

Subgroup analysis and meta-regression showed that duration per muscle group and monitoring time points were the main sources of heterogeneity. Regarding duration per muscle group, the difference between groups was marginally significant (P_m_ = 0.06). Specifically, prolonged intervention (> 5 min) showed a significant, moderate positive effect size (g = 0.60, 95% CI 0.03 to 1.17, *p* = 0.04). This suggests that excessive treatment time may lead to increased DOMS scores. Meta-regression further confirmed this trend. The results showed a significant positive linear correlation between duration and effect size (β = 0.43, *p* = 0.032). This explained 14.79% of the heterogeneity between studies. This indicates that as single-session treatment time increases, the effect of alleviating DOMS decreases. Regarding monitoring time points, we observed significant differences between groups (P_m_ = 0.04). The effect size was largest at 72 h post-exercise (g = 1.23, *p* = 0.01). Consistently, meta-regression indicated that monitoring time was a significant predictor of effect size (*p* = 0.026). It explained 26.23% of the heterogeneity. In contrast, neither PT frequency (P_m_ = 0.57, regression *p* = 0.565) nor exercise mode (P_m_ = 0.64, regression *p* = 0.282) showed significant moderating effects on DOMS (Table 7, Fig. 9).

**Table 7:** Subgroup Analysis of Percussive Therapy's Effect on Post-Exercise DOMS

Subgroup	K(N)	Hedges'g	95%CI	P_d_	Q	I^2^(%)	P_m_
Duration of treatment							0.06
≤ 2.5min	4(82)	-0.29	-1.14 ~ 0.56	0.50	0.00	93.73	
2.5-5min	6(104)	-0.17	-0.50 ~ 0.15	0.30	0.97	0.00	
> 5min	7(101)	0.60	0.03 ~ 1.17	0.04	0.00	85.89	
Frequency							0.57
< 50Hz	9(104)	0.04	-0.43 ~ 0.61	0.87	0.01	61.66	
≥ 50Hz	8(189)	0.24	-0.26 ~ 0.74	0.35	0.00	91.24	
Monitoring time							0.04
0h	3(169)	0.17	-0.09 ~ 0.43	0.20	0.46	0.00	
24h	8(273)	-0.21	-0.63 ~ 0.22	0.33	0.00	69.14	
48h	4(196)	0.34	-0.29 ~ 0.97	0.29	0.00	80.32	
72h	2(107)	1.23	0.28 ~ 2.17	0.01	0.05	73.51	
Exercise Modality							0.64
Concentric	4(105)	0.02	-0.29 ~ 0.32	0.91	0.06	58.53	
Eccentric	9(104)	0.04	-0.43 ~ 0.51	0.87	0.01	61.66	
Aerobic	4(84)	0.50	-0.46 ~ 1.46	0.31	0.00	94.43	

K(N): number of included effect sizes (total number of participants); P_d_: p-value of the pooled effect size for the subgroup; Q and I^2^(%): statistics for heterogeneity; P_m_: p-value for the test of subgroup differences. N/A: stands for not applicable. NR: No relevant information available

#### Meta-regression bubble plots

See Figs. 9 and 10.

**Fig. 10:**
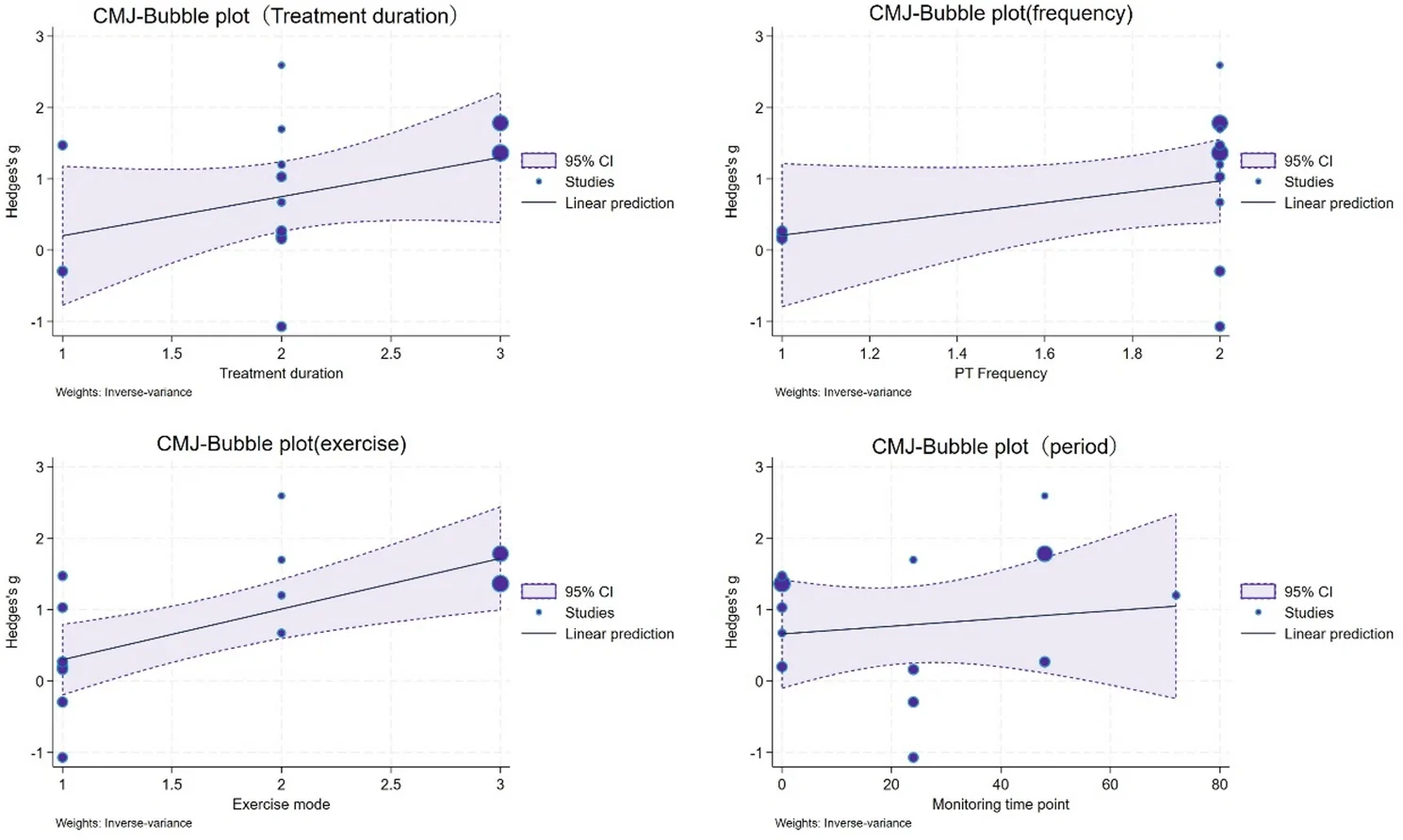
Meta-regression analysis of key variables affecting CMJ

#### Sensitivity analysis

**Fig. 11:**
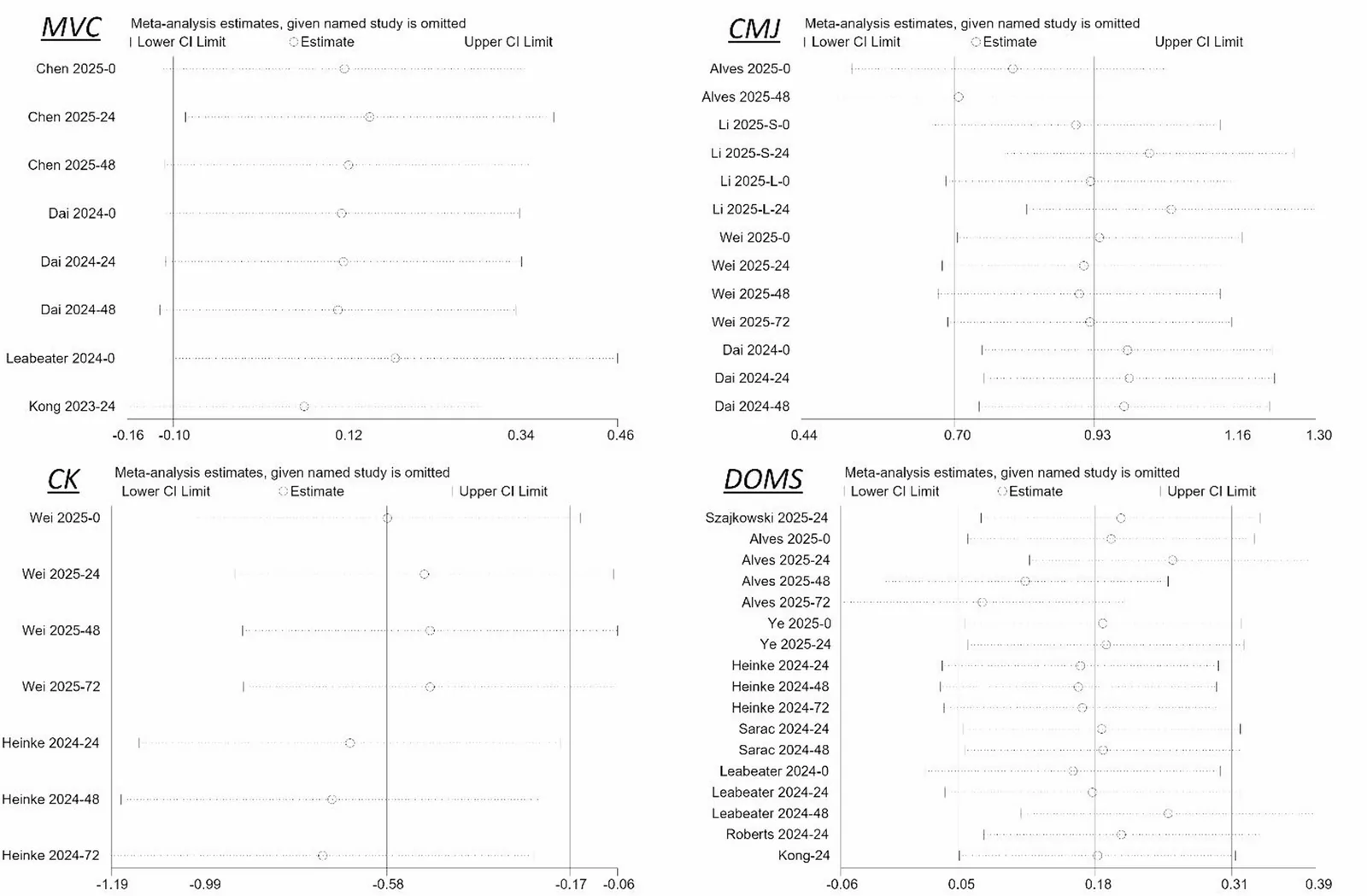
Sensitivity Analysis Results for DOMS, CK, MVC, CMJ (Stepwise Elimination Method)

Sensitivity analysis using the "leave-one-out" method confirmed that our results were highly robust. We removed individual studies one by one for all four outcomes (DOMS, CMJ, MVC, and CK). This process did not change the direction of the effect or the statistical significance of the pooled results. All recalculated 95% confidence intervals remained consistent with the original results. This indicates that a single data source did not drive the conclusions (Fig. 11).

#### Publication bias

**Fig. 12:**
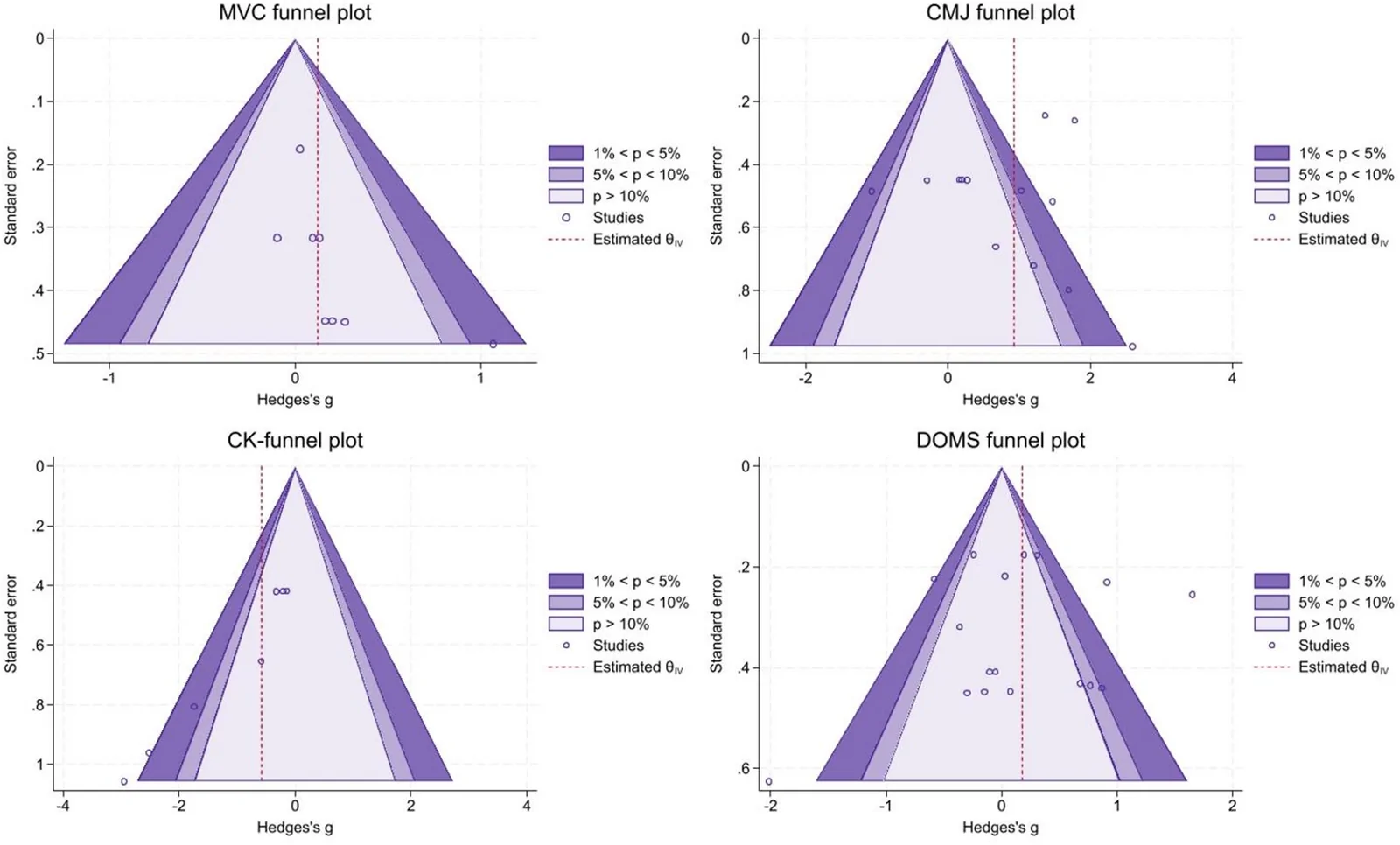
Summary of all results of the funnel plot Overall Overview

We assessed publication bias using funnel plots, Egger's test, and the "trim and fill" method. Egger's regression analysis showed no significant small-study effects for DOMS (*p* = 0.767) or CMJ (*p* = 0.357). We did not perform statistical tests for MVC and CK because the number of studies was small (k < 10). However, visual inspection showed relatively symmetrical distributions. To further verify robustness, we analyzed CMJ using Duval and Tweedie's "trim and fill" method. We did this because CMJ showed visual asymmetry. After imputing three missing studies on the left side of the funnel plot, the adjusted pooled effect size for CMJ dropped to g = 0.492 (95% CI − 0.039 to 1.023). The confidence interval crossed the line of null effect. This suggests that while PT generally promotes explosive power recovery, unpublished negative results may influence this conclusion. Therefore, practitioners should interpret this finding with caution. In contrast, MVC results remained unchanged after the "trim and fill" analysis. This demonstrates high robustness (Fig. 12).

## Discussion

### Main findings

This systematic review and meta-analysis used existing RCTs. It quantified the dose-dependent characteristics of PT in acute exercise recovery for the first time. The study revealed that PT effects are specific to the outcome and dependent on the dosage. Based on the GRADE assessment (Table 8), pooled results showed that PT significantly promoted CMJ (low certainty) and reduced CK levels (very low certainty), but did not significantly improve MVC (moderate certainty) or alleviate DOMS (very low certainty). Subgroup analysis and meta-regression identified treatment duration and vibration frequency as key regulators. Long-duration intervention (> 5 min per muscle group) was necessary to reverse neuromuscular decline (CMJ). However, it carried a risk of inducing mechanical pain sensitization, thus exacerbating DOMS. High-frequency stimulation (≥ 50 Hz) was the threshold condition for clearing metabolic markers (CK). This finding challenges the traditional logic that "improved sensation means functional recovery." It suggests that muscle rheological remodeling, pain modulation, and metabolic clearance follow different dose–response mechanisms in mechanobiology [44, 45]. At the same time, the overall interpretation must remain cautious because several pooled outcomes were informed by a relatively small number of studies, some analyses showed substantial heterogeneity, and the apparent benefit for CMJ may also have been influenced by small-study or publication bias. Therefore, the present results do not support PT as a universal recovery tool, but rather support a more selective and evidence-calibrated application pending confirmation from larger, preregistered, high-quality trials.

**Table 8:** GRADE Summary of Findings for Main Outcomes

Outcome indicators	Risk of Bias	Inconsistency	Indirectness	Imprecision	Publication bias	Certainty assessment
MVC	Not serious ↔	I^2^ = 0.00% ↔	Not serious ↔	Insufficient sample size (all under 400 participants) ↓	Symmetrical funnel chart ↔	⨁⨁⨁◯ Moderate
CMJ	Not serious ↔	I^2^ = 76.03% ↓	Not serious ↔		p = 0.357 ↔	⨁⨁◯◯ Low
CK	Not serious ↔	I^2^ = 59.96% ↓	Not serious ↔		Asymmetric funnel chart ↓	⨁◯◯◯ Very Low
DOMS	↓	I^2^ = 83.31% ↓	Not serious ↔		p = 0.767 ↔	⨁◯◯◯ Very Low

Risk of Bias: Not downgraded for MVC, CMJ, and CK because these outcomes were assessed using objective measures, making major bias from lack of blinding unlikely; downgraded by one level for DOMS because it was a subjective, participant-reported outcome and therefore more susceptible to bias. ↓ indicates a reduction by one level; ↔ indicates no reduction; I^2^ indicates heterogeneity

### Strength recovery

Long-duration intervention (> 5 min) showed superior benefits for explosive power recovery. This finding extends the conclusions of Konrad et al. [15]. Unlike their study, which only confirmed PT prevents force deficits in non-fatigued states, our results apply to fatigued states. From a neural control perspective, PT-induced recovery is not just an immediate effect of the Tonic Vibration Reflex (TVR). It involves a more lasting neural facilitation process. Continuous vibration stimulates Ia afferent fibers in muscle spindles. This increases motor unit recruitment rates and synchronization [46, 47]. Notably, short interventions cause a transient spinal reflex [48]. In contrast, long-duration stimulation may induce synaptic plasticity in the corticospinal pathway. This occurs through "Hebbian learning" mechanisms [49, 50]. This cumulative stimulation induces sustained neural excitability in fatigued muscles. It effectively offsets the decline in Central Drive caused by EIMD [47]. This process resembles Post-activation Potentiation (PAP) [51].Optimized neural recruitment is crucial for CMJ recovery. CMJ performance relies heavily on neural firing rates [52]. Beyond neural adaptation, long-duration intervention benefits CMJ through rheological remodeling of the Muscle–Tendon Unit (MTU). This process restores the energy storage capacity of the Series Elastic Component (SEC). It effectively counteracts the inhibition of the Stretch–Shortening Cycle (SSC) caused by EIMD [53, 54]. Mechanically, continuous load fits the thixotropy model. It significantly reduces tissue viscosity [11]. The thermodynamic threshold effect is also critical. Only long-duration intervention generates enough heat to increase nerve conduction velocity [55]. This confirms that sufficient dosage is central to remodeling SSC function [56]. In contrast, PT had limited effects on MVC recovery. Intervention-induced tissue relaxation and thixotropy temporarily reduce passive stiffness in the muscle–tendon unit. This causes force transmission delays and internal shortening during isometric contraction. It also triggers residual force depression. Ultimately, these factors offset the neural benefits [57, 58]. Overall, these findings support the outcome specificity of PT for strength recovery, with possible benefits for dynamic/explosive performance but no clear advantage for maximal static strength under the current evidence base.

### Biochemical markers

This study found that PT may reduce serum CK levels after exercise, with subgroup findings suggesting that 2.5–5 min per muscle group combined with high-frequency stimulation (≥ 50 Hz) may be associated with lower CK values. However, this outcome was supported by very low-certainty evidence and only a small number of studies; therefore, these findings should be interpreted cautiously. A possible explanation is that PT may enhance local fluid exchange and the mechanical muscle-pump effect, thereby facilitating the transport of muscle-derived proteins such as CK [22, 59–62]. However, these mechanisms were not directly assessed in the included studies and should be regarded as plausible rather than confirmed explanations. In addition, our subgroup findings suggested that continuous treatment for more than 5 min did not provide further CK reduction, which may indicate a plateau in transport efficiency or limited lymphatic responsiveness under prolonged loading [63–65]. The possibility that excessive mechanical stimulation may contribute to secondary micro-trauma should also be considered hypothetical rather than established [66]. Overall, the apparent benefit of PT for CK clearance remains preliminary and requires confirmation in larger, high-quality trials with standardized mechanical parameters.

### Subjective pain

An interesting finding in this study is an apparent contradiction: although PT appeared to promote the clearance of muscle damage markers, it did not significantly reduce DOMS after acute exercise. Moreover, prolonged intervention even seemed to increase pain, peaking at 72 h post-exercise. This "non-significant finding" diverges from recent positive results by Alves et al. [17], but is more consistent with Roberts et al. [18], who suggested that PT may provide only transient analgesia without altering the course of DOMS. The substantial heterogeneity (83.31%) indicates that the effect of PT on pain is unlikely to be linear and may depend on the pathological state and intervention dosage. A possible explanation is that, during the acute injury phase (24–72 h), inflammatory mediators may lower the threshold for mechanical pain provocation [67]. Under these conditions, short-duration stimulation may still induce temporary analgesia via the gate control mechanism [68], whereas prolonged stimulation may exceed this sensitized threshold and be perceived as additional nociceptive input [69, 70]. Excessive mechanical loading may also contribute to secondary micro-trauma, particularly because PT devices provide constant output without tactile adjustment [66]. Unlike traditional manual therapy, it cannot dynamically adjust based on tissue stiffness [22]. This constant output easily triggers excessive stimulation during the sensitization phase. When applied to highly sensitized tissue, it exacerbates microvascular leakage and tissue edema [71]. Additionally, the lack of standardized pressure application was a source of high heterogeneity in this study [72]. Therefore, the present findings do not exclude a short-lived analgesic effect of PT, but they do not support a consistent benefit for DOMS relief under the current evidence base.

### Limitations

This study was the first to quantify the effects of PT on post-exercise strength recovery via meta-analysis. It revealed a “dose-outcome separation” between biochemical clearance and pain modulation. However, readers must consider the following methodological limitations. First, although impact frequency and treatment duration were extracted, most included RCTs did not report amplitude in detail. This made it impossible to define the complete mechanical boundaries of the optimal dosage. In addition, not all included RCTs were preregistered, which raises concerns about selective reporting and may have contributed to heterogeneity. Second, physical interventions have obvious sensory characteristics; therefore, double-blind designs were difficult to implement. This may introduce bias, especially for subjective pain scores such as VAS, which are susceptible to expectation and placebo effects. Third, the evidence for key biochemical markers was limited, as only two studies reported CK levels after intervention. Fourth, most participants were healthy young males. Because human soft tissue damping correlates with body fat percentage, subcutaneous fat layers may weaken high-frequency mechanical wave transmission [73]. The limited data on females and elite athletes restrict the external validity of our conclusions for broader populations. Finally, Cohen’s κ values for study selection, PEDro scoring, and RoB 2 assessment were calculated post hoc from archived independent reviewer records. These values should therefore be interpreted as descriptive indicators of reviewer agreement rather than prespecified methodological outcomes. Future studies should preregister their protocols, standardize the reporting of mechanical parameters, and include objective physiological markers to develop more precise recovery protocols.

## Conclusion

This systematic review and meta-analysis suggests that PT may have outcome-specific effects during acute post-exercise recovery. PT may promote CMJ recovery and may reduce CK levels, but current evidence does not show clear benefits for MVC recovery or DOMS relief. Dose-related findings were observed: longer treatment duration (> 5 min per muscle group) appeared more favorable for CMJ recovery, whereas 2.5–5 min protocols combined with higher frequency (≥ 50 Hz) appeared more favorable for CK reduction. However, these dose patterns were derived from exploratory subgroup and meta-regression analyses and should not be interpreted as definitive clinical thresholds. Prolonged intervention may be associated with higher DOMS scores in sensitized tissue, but this finding also requires confirmation. Therefore, PT should not be regarded as a universal recovery strategy; rather, these preliminary dosage patterns may inform individualized recovery planning while awaiting larger, preregistered, high-quality RCTs with standardized mechanical-parameter reporting.

## Supplementary Information

Below is the link to the electronic supplementary material.

Supplementary Material 1.

## Data Availability

All data extracted or analyzed during this study were included in this published article and its supplementary information files.
