# Supplementary material for: Effects of percussive therapy dosages on recovery from acute exercise-induced muscle damage: a systematic review and meta-analysis
Source: Chiropr Man Therap. 2026 Jun 12;34:34. doi: 10.1186/s12998-026-00657-9 (PMC13488264; doi:10.1186/s12998-026-00657-9)
Supplement: Supplementary file 1 [file 12998_2026_657_MOESM1_ESM.docx]

# 1 Supplementary Table S1

Table S1 Number of hits for the complete search strategy in the databases

| Database | Complete Search Strategy | Hits Nov 26, 2025 |
| --- | --- | --- |
| PubMed | #1 ("percussive therapy"[Title/Abstract] OR "percussion therapy"[Title/Abstract] OR "percussive massage"[Title/Abstract] OR "percussion massage"[Title/Abstract] OR "massage gun"[Title/Abstract] OR "vibratory massage"[Title/Abstract] OR "percussion device"[Title/Abstract] OR "handheld vibration"[Title/Abstract] OR "local vibration"[Title/Abstract] OR "vibration therapy"[Title/Abstract] OR "percussive massage device"[Title/Abstract] OR "percussion vibration"[Title/Abstract] OR "theragun"[Title/Abstract] OR "hypervolt"[Title/Abstract] OR "hyperice"[Title/Abstract] OR "percussion massage gun"[Title/Abstract])  #2 ("Exercise"[MeSH Terms] OR ("Post-Exercise Recovery"[MeSH Terms] OR "Post-Exercise Recovery Techniques"[MeSH Terms]) OR "Resistance Training"[MeSH Terms] OR "Recovery of Function"[MeSH Terms] OR ("Muscle Strength"[MeSH Terms] OR "Muscle Strength Dynamometer"[MeSH Terms]) OR ("recovery"[Title/Abstract] OR "muscle"[Title/Abstract] OR "DOMS"[Title/Abstract] OR "pain"[Title/Abstract] OR "ROM"[Title/Abstract] OR "range of motion"[Title/Abstract] OR "strength"[Title/Abstract] OR "muscle stiffness"[Title/Abstract] OR "fatigue"[Title/Abstract] OR "performance"[Title/Abstract] OR "EIMD"[Title/Abstract] OR "creatine kinase"[Title/Abstract] OR "CK"[Title/Abstract] OR "flexibility"[Title/Abstract] OR "jump"[Title/Abstract] OR "CMJ"[Title/Abstract] OR "power"[Title/Abstract] OR "agility"[Title/Abstract] OR "MVC"[Title/Abstract] OR "shear modulus"[Title/Abstract] OR "myotonometry"[Title/Abstract])  #3 ("Randomized Controlled Trial"[Publication Type] OR "Randomized Controlled Trials as Topic"[MeSH Terms] OR "Controlled Clinical Trial"[Publication Type] OR ("Randomized Controlled Trial"[Title/Abstract] OR "clinical trial"[Title/Abstract] OR "placebo"[Title/Abstract] OR "randomly"[Title/Abstract] OR "groups"[Title/Abstract] OR "crossover"[Title/Abstract] OR "rct"[Title/Abstract])  #4 (humans[Filter]) AND (2000:2025[pdat])  #5 #1 AND #2 AND #3 AND #4 | 142 |
| Web of Science | A comprehensive literature search was conducted in the Web of Science (Core Collection) database on November 26, 2025. The search strategy combined concepts for the intervention, outcomes, and study design using Boolean operators. The specific search string used was: (TS=("percussive therapy" OR "percussion therapy" OR "percussive massage" OR "percussion massage" OR "massage gun" OR "myofascial gun" OR "vibration gun" OR "percussion gun" OR "vibratory massage" OR "percussion device" OR "Handheld vibration" OR "local vibration" OR "vibration therapy" OR "percussion massager" OR "percussive massage device" OR "vibratory percussion" OR "percussion vibration" OR "rapid percussion" OR "deep tissue percussion" OR "Theragun" OR "Hypervolt" OR "Hyperice" OR "percussive massage gun" OR "percussion massage gun" OR "handheld percussive massager" OR "percussion vibrator")) AND (TS=("Exercise" OR "Post-Exercise Recovery" OR "Resistance Training" OR "Exercise Test" OR "Recovery of Function" OR "Muscle Strength" OR "Sports" OR "recovery" OR "muscle" OR "DOMS" OR "pain" OR "ROM" OR "range of motion" OR "strength" OR "muscle stiffness" OR "fatigue" OR "performance" OR "EIMD" OR "creatine kinase" OR "CK" OR "flexibility" OR "jump" OR "CMJ" OR "power" OR "agility" OR "MVC" OR "shear modulus" OR "myotonometry")) AND (TS=(randomized controlled trial OR clinical trial OR placebo OR randomly OR groups OR crossover OR RCT)). The results were further refined by restricting the Document Type to "Article", which yielded a final total of 181 records. | 181 |
| Cochrane library | #1 (("percussive therapy" OR "percussion therapy" OR "percussive massage" OR "percussion massage" OR "massage gun" OR "myofascial gun" OR "vibration gun" OR "percussion gun" OR "vibratory massage" OR "percussion device" OR "Handheld vibration" OR "local vibration" OR "vibration therapy" OR "percussion massager" OR "percussive massage device" OR "vibratory percussion" OR "percussion vibration" OR "rapid percussion" OR "deep tissue percussion" OR "Theragun" OR "Hypervolt" OR "Hyperice" OR "percussive massage gun" OR "percussion massage gun" OR "handheld percussive massager" OR "percussion vibrator")):ti,ab,kw  #2 (("Exercise" OR "Post-Exercise Recovery" OR "Resistance Training" OR "Exercise Test" OR "Recovery of Function" OR "Muscle Strength" OR "Sports" OR "recovery" OR "muscle" OR "DOMS" OR "pain" OR "ROM" OR "range of motion" OR "strength" OR "muscle stiffness" OR "fatigue" OR "performance" OR "EIMD" OR "creatine kinase" OR "CK" OR "flexibility" OR "jump" OR "CMJ" OR "power" OR "agility" OR "MVC" OR "shear modulus" OR "myotonometry")):ti,ab,kw  #3 #1 AND #2 | 428 |
| Embase | (('percussive therapy':ti,ab,kw OR 'percussion therapy':ti,ab,kw OR 'percussive massage':ti,ab,kw OR 'percussion massage':ti,ab,kw OR 'massage gun':ti,ab,kw OR 'vibratory massage':ti,ab,kw OR 'percussion device':ti,ab,kw OR 'handheld vibration':ti,ab,kw OR 'local vibration':ti,ab,kw OR 'vibration therapy':ti,ab,kw OR 'percussive massage device':ti,ab,kw OR 'percussion vibration':ti,ab,kw OR 'theragun':ti,ab,kw OR 'hypervolt':ti,ab,kw OR 'hyperice':ti,ab,kw OR 'percussion massage gun':ti,ab,kw) AND ('exercise'/exp OR 'exercise recovery'/exp OR 'resistance training'/exp OR 'exercise test'/exp OR 'convalescence'/exp OR 'muscle strength'/exp OR 'sport'/exp OR 'recovery':ti,ab,kw OR 'muscle':ti,ab,kw OR 'doms':ti,ab,kw OR 'pain':ti,ab,kw OR 'rom':ti,ab,kw OR 'range of motion':ti,ab,kw OR 'strength':ti,ab,kw OR 'muscle stiffness':ti,ab,kw OR 'fatigue':ti,ab,kw OR 'performance':ti,ab,kw OR 'eimd':ti,ab,kw OR 'creatine kinase':ti,ab,kw OR 'ck':ti,ab,kw OR 'flexibility':ti,ab,kw OR 'jump':ti,ab,kw OR 'cmj':ti,ab,kw OR 'power':ti,ab,kw OR 'agility':ti,ab,kw OR 'mvc':ti,ab,kw OR 'shear modulus':ti,ab,kw OR 'myotonometry':ti,ab,kw) AND ('randomized controlled trial':it OR 'randomized controlled trial (topic)'/exp OR 'controlled clinical trial':it OR 'randomized controlled trial':ti,ab,kw OR 'clinical trial':ti,ab,kw OR 'placebo':ti,ab,kw OR 'randomly':ti,ab,kw OR 'groups':ti,ab,kw OR 'crossover':ti,ab,kw OR 'rct':ti,ab,kw) AND [humans]/lim AND ('article'/it OR 'conference abstract'/it OR 'clinical trial'/it)) | 297 |

# 2 Supplementary S2

**S2 Study Selection Consistency and Cohen’s κ**

## 2.1 Title/Abstract Screening (Records Screened = 629)

- Independent 2×2 table (L.L. vs T.L.): both include = 49; L.L. include / T.L. exclude = 4; L.L. exclude / T.L. include = 5; both exclude = 571 (total n = 629; a + b + c = 58, consistent with the PRISMA flowchart).
- Observed agreement: Po = (49+571)/629 ≈ 0.9857
- Expected agreement: Pe ≈ 0.8444
- Cohen’s κ ≈ 0.908 (more precisely 0.9081)
- Approximate standard error: SE ≈ 0.030
- 95% CI: κ ≈ 0.849–0.968
- Z ≈ 29.9 (p ≪ 0.001)
- Interpretation (Landis & Koch): κ ≈ 0.908, indicating almost perfect agreement.

## 2.2 Full-Text Screening (Reports Assessed = 45)

- Independent 2×2 table (L.L. vs T.L.): both include = 10; L.L. include / T.L. exclude = 1; L.L. exclude / T.L. include = 1; both exclude = 33 (total n = 45; a + b + c = 12, consistent with the PRISMA flowchart).
- Observed agreement: Po = (10+33)/45 ≈ 0.9556
- Expected agreement: Pe ≈ 0.6306
- Cohen’s κ ≈ 0.880 (more precisely 0.8797)
- Approximate standard error: SE ≈ 0.083
- 95% CI: κ ≈ 0.717–1.000 (upper bound truncated at 1.00 as customary when the normal approximation slightly exceeds 1)
- Z ≈ 10.6 (p ≪ 0.001)
- Interpretation (Landis & Koch): κ ≈ 0.880, indicating almost perfect agreement.

## 2.3 Inter-Rater Agreement Results for Each ROB2 Domain

| Domain | Simple Agreement Rate | Cohen’s κ | Strength of Agreement |
| --- | --- | --- | --- |
| D1 (Randomization process) | 83.3% (10/12) | 0.65 | Substantial |
| D2 (Deviations from intended interventions) | 91.7% (11/12) | 0.78 | Substantial |
| D3 (Missing outcome data) | 100% (12/12) | 1.00 | Almost Perfect |
| D4 (Measurement of the outcome) | 83.3% (10/12) | 0.65 | Substantial |
| D5 (Selection of the reported result) | 100% (12/12) | 1.00 | Almost Perfect |

**Table note:** RoB 2 judgments were calculated separately for each domain across the 12 included studies. Cohen’s κ ranged from 0.65 to 1.00, indicating substantial to almost perfect agreement.

## 2.4 Methodological Quality Assessment (PEDro Scale, Total Items Evaluated = 132)

- Independent 2×2 table for item scores (L.L. vs T.L.): both assigned "1" (Yes) = 88; L.L. assigned "1" / T.L. assigned "0" = 2; L.L. assigned "0" / T.L. assigned "1" = 2; both assigned "0" (No) = 40 (total items n = 12 studies × 11 items = 132). Final consensus resolved to 90 "1"s and 42 "0"s, matching the final PEDro summary table.
- Observed agreement: Po = (88+40)/132 ≈ 0.9697
- Expected agreement: Pe = [(90/132) × (90/132)] + [(42/132) × (42/132)] ≈ 0.4649 + 0.1012 ≈ 0.5661
- Cohen’s κ ≈ 0.930 (more precisely 0.9298)
- Approximate standard error: SE ≈ 0.034
- 95% CI: κ ≈ 0.863–0.997
- Z ≈ 27.3 (p ≪ 0.001)
- Interpretation (Landis & Koch): κ ≈ 0.930, indicating almost perfect agreement.

# 3 Supplementary Figure S3

**S3 Sensitivity Analysis Results for DOMS, CK, MVC, CMJ**

## 3.1 DOMS

## 3.2 CK

## 3.3 MVC

## 3.4 CMJ
